# Comparison of 75 mg versus 150 mg aspirin for the prevention of preterm preeclampsia in high-risk women at a tertiary level hospital: study protocol for a randomized double-blind clinical trial

**DOI:** 10.1186/s13063-024-08520-z

**Published:** 2024-10-15

**Authors:** Upma Saxena, Abhishek Lachyan, Chanchal Goyal, Garima Kapoor, Kavita Agarwal, Sidarrth Prasad

**Affiliations:** 1 Department of Obstetrics and Gynecology, VMMC & Safdarjung Hospital, New Delhi, 110029 India; 2 Indian Council of Medical Research (ICMR), New Delhi, 110029 India; 3 University of Texas Southwestern Medical Center, Dallas, TX USA

**Keywords:** Aspirin prophylaxis, Clinical trial, Fetal outcomes, High-Risk women, Hypertensive disorders of pregnancy, Maternal outcomes, Obstetric care, Pre-Eclampsia, Preterm pre-Eclampsia, Evidence-Based practice

## Abstract

**Background:**

Hypertensive disorders of pregnancy (HDP) pose significant risks to maternal and fetal health, with substantial mortality and morbidity rates globally, particularly in developing countries. Pre-eclampsia (PE) accounts for a notable portion of maternal morbidity and mortality, with varied prevalence across regions within countries like India. Despite advancements, disparities in healthcare access persist, influencing outcomes. PE not only affects maternal health during pregnancy but also predisposes women to long-term cardiovascular complications, emphasizing the need for early screening and preventive measures.

**Methods:**

This prospective randomized double-blind clinical trial aims to compare the efficacy and safety of 75 mg versus 150 mg aspirin for preventing preterm pre-eclampsia in high-risk women. Screen-positive women aged 18–45 years with singleton pregnancies between 12 and 16 weeks of gestational age will be enrolled. They will be randomized in a 1:1 ratio to receive either 75 mg or 150 mg of aspirin nightly until 37 weeks of pregnancy or earlier if preterm pre-eclampsia develops. Feto-maternal outcomes, including preterm pre-eclampsia incidence and neonatal and maternal complications, will be assessed. The sample size calculation based on expected proportions of preterm pre-eclampsia in both groups indicates a total of 370 participants (185 per group) accounting for 20% attrition.

**Discussion:**

This prospective randomized double-blind clinical trial aims to compare the effectiveness and safety of two doses of aspirin (75 mg vs 150 mg) in preventing preterm pre-eclampsia in high-risk women. The potential implications of this study are significant, including the optimization of aspirin prophylaxis, the development of evidence-based guidelines, and comprehensive assessment of maternal and fetal outcomes. In conclusion, the results of this study have the potential to significantly impact clinical practice by enhancing maternal and perinatal health outcomes and contributing to evidence-based obstetric care.

**Trial registration:**

Clinical Trials Registry-India CTRI/2023/12/060983. Trial was registered prospectively on 29 December 2023. Acknowledgement Number REF/2023/12/076358. https://acrobat.adobe.com/id/urn:aaid:sc:AP:15870322-f1f4-4460-900c-6e056ab83a44.

**Supplementary Information:**

The online version contains supplementary material available at 10.1186/s13063-024-08520-z.

## Introduction

Hypertensive disorders of pregnancy (HDP) are a major health problem worldwide, accounting for 500, 000 fetal deaths and 70,000 maternal deaths, and of these, every year, 40,000 maternal deaths are from developing countries [1]. Globally hypertensive disorders of pregnancy and chronic hypertension affect approximately 10% and 1.5% of pregnant women, respectively, with serious fetal and maternal implications [2].

Pre-eclampsia (PE) affects 5–7% of all pregnancies and along with eclampsia is the major contributor to maternal morbidity and 10–15% of maternal mortality and also perinatal mortality. In India, the prevalence of HDP and pre-eclampsia is 9% and 3–6%, respectively, contributing to 18.35% of maternal morbidity [3].

The highest prevalence from India is reported in southern India (Lakshadweep—65.88% and Kerala—61.16%) and western India (Goa—63.38%) and lowest in Eastern India (Nagaland 2.36%), which may be due to the difference in antenatal coverage of these states. A study from South India reported that hypertension complicates 10.3% of pregnancies in northwest Karnataka [4]. There are disparities within India in access to health care and death rates due to HDP, which vary across states and also between urban and rural populations [5].

A systematic review, including mostly cross-sectional studies from southern India, showed high pooled prevalence of HDP of 11% (95% confidence interval, 5–17%), i.e., 1 out of 11 women suffers from it. Hence, it is the need of the hour to implement early screening for HDP through focused attention of policy makers and healthcare professionals [3]. An Indian study similarly found that the incidence of eclampsia is 1.5%, and there is no reduction in it and perinatal mortality rate over the last few decades, although there is a slight decline in maternal mortality [6].

Delivery resolves most signs and symptoms of PE but persistent postpartum pre-eclampsia is associated with risk for short-term and long-term cardiovascular and cerebrovascular morbidity. These women are predisposed to an increased risk of peripartum cardiomyopathy (PPCM) that can progress to chronic heart failure, which may lead to death.

Women with a history of HDP especially high diastolic blood pressure have two to three times higher risk of cardiovascular mortality compared to that of women with normal blood pressure during pregnancy, needing individualized cardiovascular follow-up to reduce their risk of mortality due to cardiovascular event [7]. PE is also associated with a fourfold increase in future heart failure and twofold increase in coronary heart disease, stroke, and death due to CHD [8].

Multiple episodes of pre-eclampsia and its increasing severity escalates future risk of cardiovascular disease. A study demonstrated that the odds of a fatal outcome from cardiovascular disease were greater than the odds of diagnosis which meant that women may die from the sequelae even without diagnosis of cardiovascular disease. Later in life, these women had 4.7-fold, fourfold, and threefold increased risk for subsequent end-stage renal disease, stroke, and vascular dementia, respectively [9]. It has been advocated that there is an urgent need for uniform guidelines and their implementation to improve the health of women with a history of PE. This can be done by making the public aware about the cardiovascular risk associated with it and by promoting a cardiovascular prevention program [10].

PE is a double-edged sword and adversely affects the newborn as well, leading to increased neonatal morbidity and mortality which may be due to iatrogenic prematurity, fetal growth restriction (FGR), and abruptio placentae [11]. An increased incidence of bronchopulmonary dysplasia and retinopathy of prematurity has also been reported in neonates due to extreme prematurity. About 80% of pre-eclampsia cost is due to preterm pre-eclampsia, leading to iatrogenic prematurity (≤ 37 weeks). The cost burden of pre-eclampsia per infant is estimated to range from $150,000 at 26 weeks gestational age reducing to $1311 at 36 weeks gestational age [12]. Children born to women with pre-eclampsia in their teenage years and beyond have an increased risk of cardiovascular disease and pulmonary hypertension [11].

The various clinical risk factors for PE are teenage pregnancy, primigravida, multiple gestation, history of diabetes, pre-existing hypertension, obesity, and previous history of HDP [13, 14]. NICE recommends screening for PE based on maternal characteristics along with medical, obstetric, and family history, which has a detection of 40% and 33% for preterm and term pre-eclampsia, respectively at a screen-positive rate of 11%. First-trimester quad was done at 11–13 + 6 weeks using maternal characteristics along with MAP, PIGF, and PAPP-A with/without UTPI and has a detection rate of 76% and 38% for preterm and term pre-eclampsia, respectively, at a FPR of 10% [11].

Preterm pre-eclampsia is due to defective placentation and low-dose aspirin (LDA) when started between 12 and 16 weeks of pregnancy corrects the imbalances in the levels of thromboxane A2 and prostacyclin, maintaining adequate uteroplacental blood supply. So aspirin improves defective placentation without increasing the risks of adverse maternal and perinatal outcomes [15].

Early prediction and prevention of pre-eclampsia would greatly contribute to improved maternal and perinatal health. Prevention of iatrogenic prematurity due to preterm PE is a very cost-effective measure. Aspirin is an inexpensive and widely available drug that has the potential to safely prevent preterm PE, resulting in reduced maternal and fetal morbidity and mortality [16]. Low-dose aspirin prophylaxis to prevent preterm pre-eclampsia is now recommended till 37 weeks of pregnancy in high-risk women [13].

Although low-dose aspirin has a good maternal and fetal safety profile, some authors have reported a higher incidence of intrapartum bleeding (2.9% aspirin users vs 1.5% nonusers), postpartum hemorrhage (10.2% vs 7.8%), postpartum hematoma (0.4% vs 0.1%), and neonatal intracranial hemorrhage (0.07% vs 0.01%) in aspirin users. A higher incidence of bleeding among aspirin users was present for those who had a vaginal birth but not those who had a cesarean delivery [15].

While numerous randomized trials have investigated the prophylactic use of aspirin in the prevention of pre-eclampsia, the optimal dose still remains unclear. Prediction of preterm PE in the first trimester enables the caregiver in preventing this deadly disease using aspirin. All “at risk” women should be started with low-dose aspirin and earlier studies have used 75–81–100–150 mg [17–19]. The safety of 150 mg of aspirin needs to be validated and the minimum most effective dose for the prevention of preterm PE should be used. Hence, there is an urgent need for more evidence-based studies to resolve this dilemma about the appropriate dose of aspirin prophylaxis, weighing the benefit versus harm of aspirin.

Hence, this prospective randomized double-blind clinical trial is being carried out to compare 75 mg versus 150 mg Aspirin for the prevention of preterm pre-eclampsia in high-risk, screen-positive women.

## Summary of relevant studies

### Systematic reviews and *meta*-analyses

A systematic review including 23 randomized controlled trials (RCTs) with a total of 26,952 participants examined the impact of LDA on pre-eclampsia. The review found that aspirin dosages ranged from 50 mg/day to 150 mg/day. In participants at increased risk for pre-eclampsia, the incidence of pre-eclampsia varied from 4 to 30%. The use of LDA was significantly associated with a lower risk of pre-eclampsia, perinatal mortality, preterm birth, and fetal growth restriction (FGR). However, the review found no significant increase in postpartum or other bleeding-related harms nor long-term harms. The absolute risk reductions for pre-eclampsia with aspirin ranged from 1 to 6% in larger trials, while the reductions in perinatal morbidity ranged from 0.5% to 1.1% in the largest trials [20].

The USPSTF concluded with moderate certainty that daily low-dose aspirin (81 mg) is beneficial for reducing the risk of pre-eclampsia, preterm birth, small-for-gestational-age, and perinatal mortality in high-risk pregnant individuals. This led to their recommendation of low-dose aspirin prophylaxis for high-risk women starting after 12 weeks of gestation [14].

### Recent studies and trials

A recent study conducted in China compared the effects of a placebo versus 75 mg daily LDA on pre-eclampsia incidence. The results indicated that 10.43% of women in the LDA group and 22.52% in the control group developed pre-eclampsia. The odds ratio for developing pre-eclampsia with LDA was 0.40 (95% CI = 0.20–0.82, *P*= 0.0098), suggesting a significant benefit of aspirin prophylaxis [21].

Wright and colleagues found that aspirin reduced the length of stay in neonatal intensive care units by approximately 70%, primarily due to a decrease in the rate of iatrogenic prematurity at < 32 weeks gestation, related to early prevention of pre-eclampsia. This reduction in NICU stay was associated with implications for both short-term and long-term healthcare costs and infant survival [22].

### Effectiveness in specific populations

A multicenter, double-blind, randomized placebo-controlled trial assessed 1776 women at high risk for preterm pre-eclampsia. Participants received either 150 mg daily aspirin or placebo from 11 to 14 weeks of gestation until 36 weeks. Preterm pre-eclampsia rates were 1.6% in the aspirin group and 4.3% in the placebo group. The study concluded that low-dose aspirin effectively prevents preterm pre-eclampsia [23].

Rolnik and colleagues conducted the ASPRE trial, involving 26,941 singleton pregnancies, to screen for preterm pre-eclampsia using a combined algorithm of maternal factors and biomarkers. Women with a preterm pre-eclampsia risk > 1 in 100 were assigned to receive 150 mg daily aspirin or placebo from 11–14 weeks until 36 weeks. The study showed a 62% reduction in preterm pre-eclampsia incidence with aspirin [23].

### Gaps in current knowledge

Despite the evidence supporting aspirin’s efficacy in preventing pre-eclampsia, several uncertainties remain. One key issue is the optimal dosage and timing, as there is no clear consensus on the most effective dose. While 75 mg and 150 mg are commonly prescribed, debate persists regarding their comparative effectiveness. Additionally, the long-term effects of aspirin on infants exposed during pregnancy are underexplored, with most research focusing on short-term outcomes and leaving a gap in understanding potential neurodevelopmental and health consequences later in life. Another challenge lies in risk stratification, as identifying women at highest risk for pre-eclampsia who would benefit most from aspirin prophylaxis remains difficult, and current criteria may not fully optimize treatment outcomes.

### Research question and justification

The primary objective of this study is to evaluate the efficacy and safety of two different dosages of low-dose aspirin (LDA), 75 mg and 150 mg daily, in preventing preterm pre-eclampsia in high-risk pregnant women. While LDA has been widely used and studied, the optimal dosage remains uncertain. Previous research indicates that aspirin can lower the incidence of preterm pre-eclampsia and its complications, but there is variability in the effectiveness of different dosages and timing of initiation.

### Need for the study

This study aims to address these gaps by comparing the effectiveness of 75 mg and 150 mg daily aspirin in preventing pre-eclampsia among high-risk pregnant women. By refining dosage guidelines, this research will help tailor preventive strategies more effectively and potentially improve maternal and fetal outcomes. Additionally, it will provide valuable insights into the long-term effects of aspirin on infant health, contributing to a more comprehensive understanding of its benefits and risks.

## Aims and objectives

### Aim

To compare 75 mg versus 150 mg aspirin for the prevention of preterm preeclampsia in high-risk women.

### Objective

#### Primary objective

To compare 75 mg versus 150 mg aspirin for the prevention of preterm preeclampsia in high-risk women.

#### Secondary objectives

1. To evaluate feto-maternal outcomes in high-risk women receiving 75 mg versus 150 mg aspirin for the prevention of preterm preeclampsia.
2. To compare the side effects of 75 mg versus 150 mg aspirin during pregnancy.

#### Rationale/gaps in existing knowledge

Lack of evidence about (i) optimum dose of aspirin, 75 versus 150 mg, for the prevention of preterm PE and (ii) when to start and stop aspirin during pregnancy for the prevention of preterm PE.

##### Novelty

No international or Indian prospective randomized double-blind interventional trial comparing 75 mg versus 150 mg aspirin for the prevention of preterm PE in high-risk/screen-positive women.

##### Research question

Is aspirin in dose of 150 mg better than 75 mg in the prevention of preterm preeclampsia in high-risk women?

##### Hypothesis

Aspirin in dose of 150 mg is better than 75 mg in the prevention of preterm preeclampsia in high-risk women.

## Material and methods

Include objective-wise work plan under the following sub-headings.

A Study design—prospective randomized double blind clinical trial
B Study site—Department of Obstetric & Gynecology, Pathology, Biochemistry & SPM**,** VMMC & Safdarjung Hospital, New Delhi
C Methods—(e.g., PICO)

### Population

All women 18–45 years attending ANC and Fetal Medicine OPD of VMMC & Safdarjung Hospital between 11 and 13 + 6 weeks of gestational age will be screened for high risk for preterm PE using the first-trimester quadruple testing (1 T Quad). Women with a risk for preterm PE 1:100 and greater will be labeled as screen positive or high risk. 1 T Quad will be done using Perkin Elmer LifeCycle 7.0 software on Victor 2D Instrument (Time Resolved Fluorescence—DELFIA).

### Inclusion criteria

Women 18–45 years of age with singleton pregnancy 12–16 weeks of POG were found to be screen-positive for preterm preeclampsia in the first trimester by 1 T Quad test.

### Exclusion criteria

- Hypersensitivity to aspirin or other NSAIDs
- Active peptic ulcer disease
- Active bleeding in pregnancy:
  1. Threatened miscarriage
  2. Expanding retroplacental hematoma
  3. APH
- Severe liver disease
- Mother on anticoagulant or anti-platelet drugs
- Underlying bleeding disorder in mother:
  1. Von Willebrand disease
  2. Immune thrombocytopenia purpura (ITP)
  3. Factor VIII deficiency (hemophilia)
  4. Severe gestational thrombocytopenia (platelet count < 100,000 × 10.^9^/L)
- Fetus diagnosed with malformation or aneuploidy

### Intervention

Screen-positive women fulfilling the criteria will be enrolled after written informed consent.

### Randomization, allocation, and blinding

Women enrolled will be randomized using a randomization chart which will be generated with the help of computer-generated random numbers. Participants will be randomized in a ratio of 1:1 to either receive 75 mg or 150 mg aspirin at night from 12 to 16 weeks of pregnancy. Low-dose aspirin will be administered at night with a full stomach, as it had been found to be significantly more effective in reducing the risk of pre-eclampsia and fetal growth restriction than when administered in the morning.

Trial drugs will be packed according to the randomization chart and will be delivered at the trial site. The randomization schedule will be strictly controlled, and the blinding of the trial drugs will be done by following the method of sequential number order. The medicine box will be serially numbered according to the participant’s enrolment number and will be dispensed accordingly. Participants, researchers, and managing clinicians will be blinded to the treatment. The trial will be two-armed prospective double-blind randomized control trial carried out over a period of 36 months. Unblinding during the trial will only be undertaken in the event of any serious adverse event (SAE).

To ensure the integrity of blinding in this trial, the preparation and packaging of the trial drugs will be managed by a pharmaceutical company. Here is how the process will be handled:

- *Independent blinding and packaging*

To ensure the integrity of double blinding and maintain confidentiality, we will contract a reputable pharmaceutical company to handle the blinding process. In India, we have access to 75 mg and 150 mg dosages of aspirin tablets. The pharmaceutical company will manage the preparation and packaging of these dosages:

Low-dose aspirin (75 mg): this will be provided as one 75 mg tablet in an empty gelatin capsule.

Low-dose aspirin (150 mg): this will be provided as two 75 mg tablets in an empty gelatin capsule.

Due to the larger size of the 150 mg aspirin tablet, it is not feasible to put a single tablet in a similar capsule for blinding purposes. Therefore, two 75 mg aspirin tablets will be used to represent the 150 mg dose, ensuring that both dosage forms are indistinguishable in appearance, taste, and packaging for blinding purposes.

Strict protocols will be followed to maintain the confidentiality of the medication and prevent any identifying features that could reveal the treatment allocation.

- *Randomization and control*

An independent statistician, who has no role in the drug preparation or handling, will overlook the randomization process. This statistician will create the computer-generated randomization sequence to ensure that treatment assignments are concealed. The pharmaceutical company will use this sequence to label the medications.

- *Labeling and distribution*

Labels will be designed to mask the treatment assignment from both participants and study staff, with uniform packaging that does not disclose any treatment information.

- *Monitoring and compliance*

Regular audits and checks will be performed to ensure compliance with blinding procedures and to quickly address any potential breaches. Any issues with blinding will be documented and resolved promptly.

- *Allocation sequence generation*

In our trial, the allocation sequence will be generated by an independent statistician using a validated computer program. This will ensure that the process is fully randomized and minimizes potential bias. The software used guarantees that the sequence is random and secure, preventing any influence from external factors or manipulations.

### Allocation sequence and participant enrollment procedures

#### Generation of allocation sequence

##### Responsibility

The allocation sequence will be generated by an independent statistician who is not involved in participant recruitment or intervention assignment. This approach will ensure the integrity and randomness of the allocation process.

##### Method

The sequence will be created using a computer-generated random number sequence or specialized randomization software designed for clinical trials. The allocation sequence will be kept confidential to prevent any potential bias.

#### Enrollment of participants

Participants are enrolled by our research team, including principal investigators and research coordinators/scientist. This team will be responsible for screening and enrolling participants according to the study criteria.

##### Principal investigator (PI)

PI will oversee the entire enrollment process, ensuring that the study protocols and eligibility criteria are strictly followed. PI will be responsible for the final approval of participant eligibility and informed consent.

##### Research coordinators/scientist

Research coordinators/scientist will manage the day-to-day enrollment activities, including screening potential participants, obtaining informed consent, and collecting baseline data.

##### Healthcare professionals

In some cases, healthcare professionals such as residents/physicians or nurses may assist in identifying potential participants and providing initial study information. However, the final decision on enrollment and intervention assignment will be managed by the research team.

#### Incidental support for trial participants

Incidental support for trial participants will cover travel costs and provide compensation for lost wages, ensuring they can attend study visits without financial hardship or impact on their livelihood. These measures will promote fairness and encourage full participation in the study.

#### Assignment to interventions

##### Process

Eligible participants who will provide consent will be assigned to interventions according to the pre-generated allocation sequence managed by the independent statistician.

##### Blinding

To maintain blinding, the assignment of participants to interventions will be conducted by personnel who are not involved in data collection or outcome assessment. This ensures that neither the participants nor the research staff are aware of the treatment assignments.

##### Blinding of allocation

The allocation sequence will be concealed using secure methods, such as sealed envelopes or electronic randomization systems. Access to the sequence will be restricted to authorized personnel, and it will be revealed only after enrollment and completion of the study.

#### Documentation and quality control

##### Documentation

All procedures related to the allocation sequence generation, participant enrollment, and intervention assignment will be meticulously documented. This will include records of the allocation sequence, enrollment logs, and intervention assignment details.

##### Quality control

Regular audits and reviews will be performed to ensure adherence to the study protocol and ethical standards. Any issues or discrepancies identified will be addressed promptly to maintain study integrity.

##### Study interventions (investigational products)

Group A: will receive 75 mg of aspirin at night with water starting 12–16 weeks till completed 37 weeks or earlier if onset of preterm PE.

Group B: will receive150 mg of aspirin at night with water starting 12–16 weeks till completed 37 weeks or earlier if onset of preterm PE.

#### Method of administration of investigational products

Every patient will be instructed to take one capsule at bed time starting from the time of enrolment. Aspirin will be stopped in both groups if a woman develops preterm PE, otherwise will be continued until completed at 37 weeks.

#### Counseling

All the participants will be counseled to take a capsule at night after dinner.

### Strategies to improve adherence to intervention protocols and monitoring procedures

#### Patient education and engagement

- ◦ **Detailed counseling:** Participants will receive comprehensive counseling on the significance of adhering to the study protocol, including the benefits of the intervention and the importance of consistent participation.
- ◦ **Written instructions:** Participants will be provided with detailed written instructions covering dosage, timing, and method of administration of the trial drugs. These instructions will supplement verbal guidance.

#### Adherence support

- ◦ Medication reminders: automated reminders, including text messages and phone calls, will be employed to assist participants in remembering to take their medication as prescribed.
- ◦ Adherence aids: participants will be provided with a bottle containing their medication for a specific number of weeks as per protocol. At each subsequent visit, the research team will check the remaining tablets to assess adherence, ensuring that participants have taken the prescribed medication as per the protocol. This method allows for regular monitoring of compliance and helps to ensure accurate tracking of medication intake throughout the trial.

### Regular monitoring and follow-up

- ◦ Scheduled visits: participants will attend regular follow-up visits to evaluate adherence and address any issues. During these visits, adherence will be assessed through communication and review of medication logs.
- ◦ Tablet return: participants will be asked to return unused medication at each visit. This will help researchers track missed doses and assess adherence rates.
- ◦ Laboratory tests: periodic laboratory tests will be conducted to monitor physiological changes or side effects related to the intervention.

### Addressing non-adherence

- ◦ Problem-solving: participants showing signs of non-adherence will be contacted to identify and address barriers they may be facing. Support strategies will be personalized, such as adjusting reminder schedules or offering additional help.

### Documentation and reporting

- ◦ Data analysis: adherence data will be systematically analyzed to identify trends and areas for improvement. This analysis will guide ongoing support strategies and necessary adjustments to the intervention protocol.

### Comparison

Primary objective will be to compare 75 mg versus 150 mg aspirin for the prevention of preterm preeclampsia in screen-positive high-risk women. Secondary objectives will be to evaluate fetomaternal outcomes in high-risk women receiving 75 mg versus 150 mg aspirin for the prevention of preterm preeclampsia. Participants will be followed and onset of preterm PE < 37 weeks will be recorded. In case the women develop preterm PE, the aspirin will be stopped. All participants developing PE and not developing PE women will be followed till delivery and fetomaternal outcome will be noted.

### Outcome

#### Primary outcomes

1. Number of screen-positive high-risk women taking 75 mg aspirin developing preterm PE
2. Number of screen-positive high-risk women taking 150 mg aspirin developing preterm PE

#### Secondary outcomes

- *Fetal*
  - Abortion
  - Preterm birth
  - Birth weight
  - Stillbirth
  - NICU admission
  - Apgar score <7
  - Early neonatal death
  - Intracranial bleeding
- *Maternal*
- **Intrapartum**
  - Bleeding—GI bleed
  - Gestational HT
  - Eclampsia
  - HELLP
  - Abruption
  - Acute kidney injury
  - Mode of delivery
- *Postpartum*
- Post partum hemorrhage
- HDU/ICU stay
- Hematoma formation
- Mortality

### Sample size

The sample size calculation is based on a study by Kumar (2020), according to which the expected proportion of PE in the two groups receiving 75 mg versus 150 mg aspirin was 17% and 6.5% respectively [10].

The sample size has been calculated according to the formula given by Sahai and Kurshid (1996):^74^

$$\text{Sample size}\;N=\frac{\left\{z_{\left(1-\alpha/2\right)}\sqrt{2 \ast \overline p\left(1-\overline p\right)}+z_{\left(1-\beta\right)}\sqrt{p_1\left(1-p_1\right)+p_2\left(1-p_2\right)}\right\}^2}{\left({\text{p}}_1-{\text{p}}_2\right)^2}\;where\;\widehat p=\frac{p_1+p_2}2$$

Expected proportion in the first group: *p*₁ = 0.17 (17%).

Expected proportion in the second group: *p*₂ = 0.065 (6.5%).

*p̂*: (*p*₁ + *p*₂)/2 = 0.1175.

Type I error: *α* = 0.05 (5%), ${z}_{\left(1-\alpha /2\right)}$ = 1.96, CI = 95%

Type II error: *β* = 0.20 (10%), ${z}_{\left(1-\beta \right)}$ = 0.842, power = 80%

Based on the formula and values given above, the sample size calculated per group is 146.6 ≈ 147 (total 294).

Allowing for 20% attrition, we get a sample size of 294/0.8 ≈370 (185/group).

Thus, with 80% power and 95% confidence interval, the minimum sample size required for the study is *185* in each group (total 370).

Reference for the formula:

*Sahai H, Kurshid A. Formulae and tables for the determination of sample size and power in clinical trials for testing differences in proportions for the two sample design: a review. Statistics in Medicine, 1996; 15: 1–21.*

### Implementation strategy

All women 18–45 years attending ANC and Fetal medicine OPD of VMMC & Safdarjung hospital between 11 and 13 + 6 weeks of gestational age (crown-rump length of 45–84 mm) will be screened for preterm PE using the first-trimester quadruple testing.

#### First-trimester quadruple Test (1 T Quad Test)

First trimester quadruple test includes maternal characteristic along with PAPA, PLGF, Free beta HCG, and alfa fetoprotein (AFP). Pre-eclampsia risk calculation will be done using maternal characteristics that increase the risk of pre-eclampsia, i.e., advanced maternal age, increased weight, Afro-Caribbean and South Asian racial origin, chronic hypertension, diabetes mellitus, systemic lupus erythematosus, antiphospholipid syndrome, in vitro fertilization conception, family history or personal history of pre-eclampsia, and first pregnancy. Using maternal characteristics along with MAP, PIGF, and PAPP-A with/without UTPI risk of preterm PE will be calculated.

Placental growth factor (PlGF) and pregnancy-associated plasma protein-A (PAPP-A) are significantly reduced in the first trimester and throughout the pregnancy in women that will later develop preterm pre-eclampsia with delivery < 37 weeks gestation. Of these two markers, PlGF has higher sensitivity for pre-eclampsia screening than PAPP-A. Biophysical markers such as mean arterial blood pressure (MAP) at 11–13 + 6 weeks’ gestation is higher in women that will later develop pre-eclampsia compared to unaffected pregnancies and is particularly raised in those women who develop the early preterm PE. The MAP will be calculated using two simultaneous recordings taken from both the arms. After the first measurements, wait for 1 min from the moment of cuff deflation and then a repeat set of measurements will be taken again from both arms. So a total of four measurements will be taken and the online calculator will then calculate the MAP multiple of the median (MoM), adjusted for the mother’s weight, height, race, smoking, chronic hypertension, diabetes, family history of pre-eclampsia, and previous history of pre-eclampsia. Only automated blood pressure monitors that are validated for use in pregnancy and pre-eclampsia will be used. The uterine artery pulsatility index (UTPI) will not be mandatory and if performed will be measured by transabdominal/transvaginal ultrasound examination at 11–13 + 6 weeks.

Prediction model for PE < 37 weeks using maternal factors + MAP along with PAPP-A and PIGF has a detection rate (DR) of 73% with 10% FPR. Adding uterine artery PI (UTPI) increases DR to 80% with FPR of 10%. 1 T Quad will be done using Perkin Elmer LifeCycle 7.0 software on Victor 2D Instrument (Time Resolved Fluorescence—DELFIA). PerkinElmer LifeCycle 7.0 software will generate a report in which a woman’s individual risk for developing preterm pre-eclampsia will be clearly presented and color coded (red = increased risk). The day IT Quad will be carried out will be visit 0.

Women with risk for preterm PE 1:100 and greater will be labeled as screen positive or high risk. Prior to any trial-related activity, the investigator will be given the verbal information and written information in a printed form about the trial to the participant/guardian(s) which they can read and understand. The investigator would ensure that the participant is fully informed about the aims, procedures, discomforts, and expected benefits of the trial. It will be emphasized that participation is voluntary and participants have the right to opt out of the trial at any time without any prejudice. A voluntary, signed informed consent will be obtained from the participant prior to clinical trial-related procedure.

#### Study procedure

All screen-positive women fulfilling the criteria will be enrolled at 12–16 weeks of pregnancy after signing the consent form. The required baseline investigations will be carried out and that day will be visit 1 or baseline. Previous obstetric history, pre-pregnancy weight, and past history of preeclampsia will be recorded. Weight and height will be recorded. Blood pressure will be measured on the right arm in a sitting position, and urine albumin/sugar test will be performed. After enrolment, the women will be randomized to receive 75 mg or 150 mg aspirin at night from 12 to 16 weeks of pregnancy. The drug will be dispensed to her after fulfilling all the formalities as per the protocol.

The subsequent follow-up will be at 20 weeks (visit 2), and four-weekly till 28 weeks, i.e., at 24 weeks (visit 3) and at 28 weeks (visit 4). After 28-week period of gestation, participants will be called two-weekly till 36 weeks, i.e., 30 weeks (visit 5), 32 weeks (visit 6), 34 weeks (visit7), and 36 weeks (visit 8). Participant will be called weekly after 36 weeks, i.e., 37 weeks (visit 9). Aspirin will be stopped at 37 weeks or earlier to 37 weeks if onset of preterm preeclampsia. If they do not develop PE, then they will be called weekly at 38 weeks (visit 10), 39 weeks (visit 11), and 40 weeks (visit 12). Subjects will visit the investigating site 12 times during the trial or if participant is not able to visit; a senior research fellow recruited in the project will visit her at her residence..Participants will be followed and whether they have onset of preterm PE < 37 weeks will be recorded. In case the women develop PE, the aspirin will be stopped. All participants developing PE and not developing PE women will be followed till delivery and fetomaternal outcome will be noted.

### Ethical consideration

The study was approved by the Institutional Ethics Committee(IEC) of VMMC-Safdarjung Hospital and registry in the Clinical Trial Registry of India; written consent of each enrolled participant will be obtained prior to the enrolment.

### Consent

Written, informed consent to participate will be obtained from all participants.

### Withdrawal criteria

- A participant if failed to adhere more than 80% to the requirements of aspirin intake and withdrew consent prior to the completion of the study.
- Every subject will be free to withdraw from the trial at any point in time for any reason.

The reason for withdrawal will be recorded in the CRF, dated, and signed. Efforts will be made to ascertain the reason for discontinuation.

#### Procurement of trial drugs

Both the aspirin dosages will be available in granules form in identical capsules. Both trial drugs will be packed only for research purposes by the GMP-certified company.

#### Assessment of drug compliance

The compliance of intake of IP will be monitored by assessing the approximate quantity of medicines consumed by the participants (minimum 80%). For this purpose, the participants will be instructed to return the empty container of the medicine at the time of each follow-up visit. Repeated reminders will be given over the phone or through family members by the project staff for regular intake of the medicine.

#### Treatment period

**Fig. 1:**
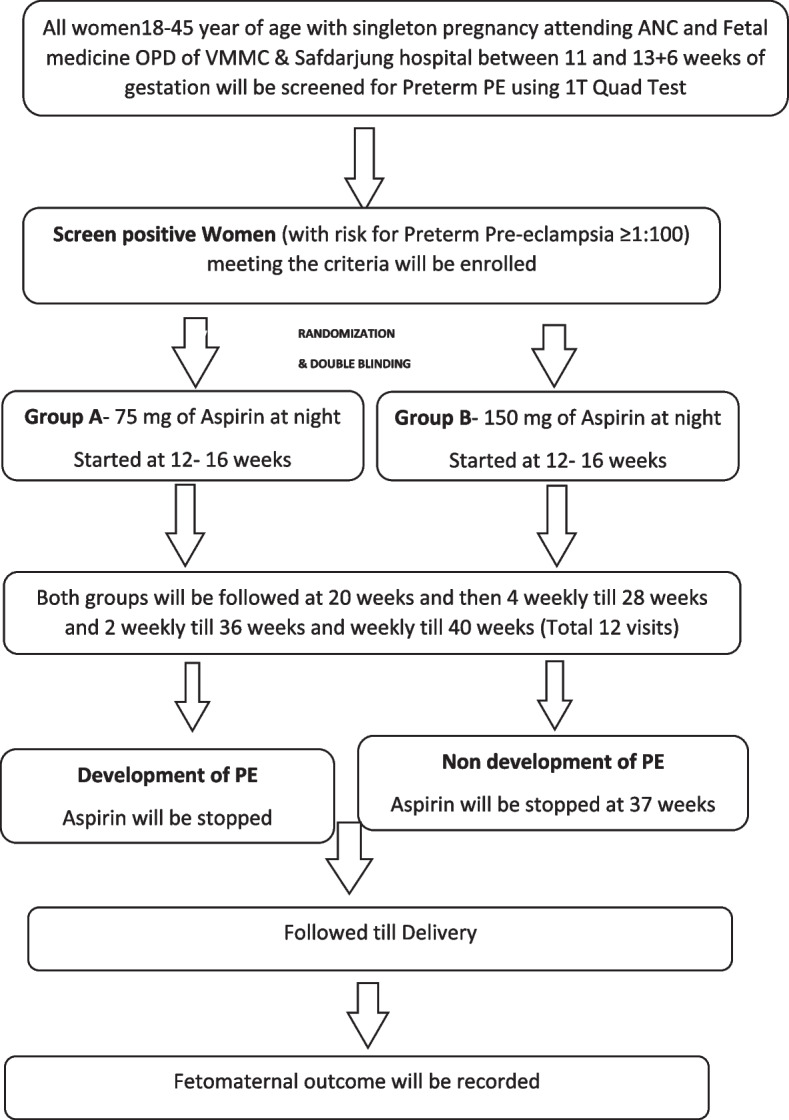
Schedule of enrolment, interventions, and assessments according to SPIRIT figure

It will be 21–25 weeks in both groups. Study participant will be recruited at 12–16 weeks. The subsequent follow-up will be at 20 weeks (visit 2) and four-weekly till 28 weeks, i.e., at 24 weeks (visit 3) and at 28 weeks (visit 4). After 28-week period of gestation, the participants will be called biweekly till 36 weeks, i.e., 30 weeks (visit 5), 32 weeks (visit 6), 34 weeks (visit7), and 36 weeks (visit 8). Participants will be called weekly after 36 weeks, i.e., 37 weeks (visit 9), 38 weeks (visit 10), 39 weeks (visit 11), and 40 weeks (visit 12). Onset of preterm PE will be recorded, aspirin will be stopped, and participants will be followed till delivery and fetomaternal outcome will be noted. In participants who do not develop preterm PE, aspirin therapy will be stopped at 37 completed weeks and will be allowed to go till 40 weeks (term) if no obstetrical indication for induction and fetomaternal outcome will be recorded. At every visit, history will be taken and they will be asked for any signs and symptoms of preeclampsia; weight and BP will be measured and urine albumin test will be done Fig. 1.

### Diagnostic criteria for preeclampsia (ACOG 2020)

#### Blood pressure

- Systolic blood pressure of 140 mm Hg or more or diastolic blood pressure of 90 mm Hg or more on two occasions at least 4 h apart after 20 weeks of gestation in a woman with a previously normal blood pressure
- Systolic blood pressure of 160 mm Hg or more or diastolic blood pressure of 110 mm Hg or more. (Severe hypertension can be confirmed within a short interval (minutes) to facilitate timely antihypertensive therapy).

#### Proteinuria

- 300 mg or more per 24-h urine collection or
- Protein/creatinine ratio of 0.3 mg/dL or more or
- Dipstick reading of 2 + (used only if other quantitative methods not available) or in the absence of proteinuria, new-onset hypertension with the new onset of any of the following:
- Thrombocytopenia: platelet count less than 100,000 10^9^/L
- Renal insufficiency: serum creatinine concentrations greater than 1.1 mg/dL or a doubling of the serum creatinine concentration in the absence of other renal disease
- Impaired liver function: elevated blood concentration of liver transaminases to twice normal concentration
- Pulmonary edema
- New-onset headache unresponsive to medication and not accounted for by alternative diagnoses or visual symptoms

#### Preeclampsia with severe features

- Systolic blood pressure of 160 mm Hg or more or diastolic blood pressure of 110 mm Hg or more on two occasions at least 4 h apart (unless antihypertensive therapy is initiated before this time)
- Thrombocytopenia (platelet count less than 100,000 10.^9^/L)
- Impaired liver function that is not accounted for by alternative diagnoses and as indicated by abnormally elevated blood concentrations of liver enzymes (to more than twice the upper limit normal concentrations) or by severe persistent right upper quadrant or epigastric pain unresponsive to medications
- Renal insufficiency (serum creatinine concentration of more than 1.1 mg/dL or a doubling of the serum creatinine concentration in the absence of other renal disease)
- Pulmonary edema
- New-onset headache unresponsive to medication and not accounted for by alternative diagnoses
- Visual disturbances

### Study procedure at follow-up

An automated reagent-strip reading device for dipstick screening for proteinuria in pregnant women will be used. If dipstick screening is positive (1 + or more), protein:creatinine ratio (PCR) will be done to quantify proteinuria in pregnant women using 30 mg/mmol as a threshold for significant proteinuria. If a participant is not able to come to the hospital for follow-up, then she will be visited at home by the research officer to ensure drug compliance and supply of study medication and also general follow-up.

### Laboratory investigations

An 8.5-ml (serum—3.5 ml, citrate—3 ml, and EDTA—2 ml) venous blood sample will be obtained after enrolment in the study. Hemogram (hemoglobin, total leucocyte count, DLC), serum TSH, ABO/Rh, VDRL, HIV, HbsAg**,** oral glucose tolerance test, liver, and kidney function tests, i.e., ALT, AST, ALP, total bilirubin, direct bilirubin, total protein, albumin, serum creatinine, urea, vitamin D levels, ionic calcium, coagulation profile, and urine routine and microscopy (urine r/m) of the enrolled participants will be carried out as baseline at the time of recruitment.

Laboratory test, i.e., CBC, LFT, KFT, S. LDH, coagulation profile, urine routine, and microscopy will be done 4 weekly. OGTT will be done first at recruitment, 24–28 weeks and at 32–34 weeks. Anomaly scan will be done at 18–20 weeks. Ultrasound with biometry and doppler will be done 4 weekly from 28 weeks in participant do not developing PE. In participants developing preterm PE, ultrasound with doppler will be done according to severity of the PE. All investigation will be done by NABH accredited lab.

#### Visit 0: Screening

- Written informed consent
- Detailed screening
- Investigational screening
- Medical history and examination
- Review of inclusion and exclusion criteria

#### Visit 1: Baseline (0 Day)

- Subjects found suitable for the study shall be enrolled.
- Medicine will be dispensed along with the drug compliance form.
- Instruction will be given to come on the next follow-up on the stipulated date.
- History of any sign and symptoms of pre-eclampsia,
- Weight,
- BP—right arm sitting
- Urine albumin/sugar

#### Successive visits

The subsequent follow-up will be for 24–28 weeks. After enrolment (visit 1), participants will be followed up at 20 weeks (visit 2) and 4 weekly till 28 weeks, i.e., at 24 weeks (visit 3) and at 28 weeks (visit 4). After 28 weeks period of gestation, the patient will be called two-weekly till 36 weeks, i.e., 30 weeks (visit 5), 32 weeks (visit 6), 34 weeks (visit 7), 36 weeks (visit 8). Participant will be called weekly after 36 weeks, i.e., 37 weeks (visit 9), 38 weeks (visit 10), 39 weeks (visit 11), and 40 weeks (visit 12).

#### Visits 2–12

- Medicine will be dispensed along with the drug compliance form.
- Instruction will be given to come on next follow up on stipulated date.
- History of any sign and symptoms of preeclampsia
- Weight
- BP—right arm sitting
- Urine albumin/sugar

Subjects will visit the investigating site 12 times during the trial or if participant is not able to visit; senior research fellow recruited in the project will visit her at her residence.

#### Standard management of any ailment arises during the study trail period

Participants registered under the trial will be instructed to avoid the use of any other drugs on their own for any ailment and will be clearly instructed to consult the treating investigator for any symptom or complaint or if they feel anything unusual. The investigator will record any medication(s) she may prescribe to alleviate their ailments. To alleviate any emergency, the use of any medication is permitted as per the discretion of the investigator. However, the same needs to be documented in the appropriate column in the case report form (CRF).

#### Drug accountability

The study drug aspirin will be kept in a secure place and will only be supplied to patients in the study under the responsibility of the investigator. A record will be kept of the study drugs dispensed. Any discrepancies between amounts dispensed and returned will be explained. Study subjects will be encouraged to maintain the count of the medication dispensed to them.

#### Drug compliance

If there is more than or equal to 80% compliance, the participant would be continued in the trial. The compliance will be assessed at each visit during the follow-up (interval depending on POG) by assessing the approximate quantity of medicines consumed by the patient. The participant will be asked to return the empty container of medicine at the time of each follow-up visit. Repeated reminders will be given over the phone or through family members and project staff for regular taking of medicine.

#### Adverse events and abnormal laboratory results

All adverse events observed or reported by patients will be recorded in the CRF with information about severity and possible relation to the study medication. Any serious adverse effects will be notified immediately to the study monitor. The investigator will report the same to the ethics committee and ICMR at the earliest. The follow-up of abnormal laboratory tests described in the CRF will be recorded accordingly.

#### Safety recording

Safety evaluation will be performed by recording clinical adverse events at randomization (baseline) and at the subsequent clinical visits. Further adverse events will be classified according to their type, severity, and possible relationship to treatment. At visit 1, the subject’s medical history will be recorded. At all visits, the vital signs, i.e., body temperature, pulse, blood pressure, will be recorded and urine for routine microscopy will be performed. Antenatal checkup will be performed as per hospital protocol and time and mode of delivery will also be as per obstetrical indication.

### Frequency and plans for auditing trial conduct

#### Project Management Group (PMG)

Frequency of meetings: the PMG will meet every 6 months to review the trial’s overall conduct. These meetings will facilitate continuous oversight and timely resolution of any issues.

#### Agenda

The meetings will focus on key performance indicators, recruitment rates, data collection progress, protocol adherence, and emerging challenges. Deviations from the protocol will be discussed, and corrective actions will be implemented as needed.

#### Documentation

Minutes from each PMG meeting will be recorded, detailing decisions, assigned actions, and follow-up deadlines. These minutes will be distributed to all PMG members.

### Trial steering group (TSG)

#### Frequency of meetings

The TSG will convene quarterly to provide strategic oversight and review the trial’s progress. Meetings will occur every 3 months to ensure alignment with study objectives and to address high-level issues.

#### Responsibilities

The TSG will review interim results, assess trial conduct, and make recommendations for protocol adjustments if necessary. They will also address any ethical or scientific concerns that arise.

#### Documentation

Detailed records, including minutes and resolutions, will be maintained from each TSG meeting to track the trial’s progress and guide decision-making.

### Independent Data Monitoring and Ethics Committee (DMC)

#### Frequency of meetings

The DMC will meet semi-annually to review interim data and ensure the trial’s integrity and participant safety. More frequent meetings may be scheduled if required based on emerging data or specific concerns.

#### Responsibilities

The DMC will review unblinded data to monitor safety, assess efficacy, and ensure ethical conduct of the trial. Recommendations regarding trial continuation, modifications, or early termination will be made as needed.

#### Documentation

The DMC will prepare and submit detailed reports on their findings and recommendations. These reports will be shared with both the TSG and PMG to inform ongoing trial management.

### Adherence to protocol and quality assurance

#### Internal audits

Regular internal audits will be performed to evaluate compliance with the study protocol, data accuracy, and overall quality. These audits will occur at least annually and may be conducted by the research team or an independent auditor.

#### External audits

The trial may undergo external audits by regulatory bodies or funding agencies to ensure adherence to regulatory and ethical standards. These audits will follow the schedule set by regulatory guidelines and funding requirements.

#### Corrective actions

Issues identified during audits or meetings will be promptly addressed. The PMG will oversee the implementation and effectiveness of corrective actions to ensure the trial’s integrity.

### Data and Safety Monitoring Board (DSMB)

A Data and Safety Monitoring Board (DSMB) will be established specifically for this trial. The DSMB will consist of independent members with relevant expertise, such as clinical researchers, biostatisticians, and ethicists. These members will have no affiliation with the trial sponsor to ensure objectivity and avoid conflicts of interest.

### Composition and independence

The DSMB will be composed of individuals who are not involved in the day-to-day management of the trial and who have no competing interests. This ensures independent oversight, with members chosen for their expertise in the specific clinical, statistical, and ethical domains pertinent to the study. The DSMB will function autonomously from the sponsor, providing unbiased assessments and recommendations.

### Role and reporting structure

The DSMB will meet periodically to review and evaluate accumulated data, with the following primary responsibilities:

- Monitor participant safety and the overall conduct of the trial.
- Assess the trial’s progress, including safety and efficacy data, where applicable.
- Make recommendations regarding the continuation, modification, or early termination of the trial based on their analysis.

The DSMB will report its findings directly to the trial sponsor and relevant regulatory authorities, maintaining its independence throughout the study ensuring transparent and objective oversight.

### Trial monitoring

The project will be monitored by the monitoring team of ICMR through regular site visits. The purpose of these visits would be to ensure strict adherence to the trial protocol, correct completion of the forms, and discuss any problems being faced by the research staff at the participating site.

### Data collection and monitoring

All history, examination, and investigations with assessment of drug compliance will be carried out at each follow-up and recorded in the CRFs. Adverse events will be noted throughout the study period. Lab investigations and ultrasound will be carried out as prehospital protocol.

#### Risk-based management

- The quality-assured investigational product (IP)will be used for the trial and labeling of IP will be properly done mentioning the manufacturing and expiring date. The IP also will be stored in a clean and safe place.
- Interim analysis will be carried out after the completion of treatment of 90 enrolled participants (45 in each group) and the result will be reported to DSMB and IEC. The investigators will check each and every data collected in CRFs on a daily basis to check the missing data mainly related to primary outcome measures.
- The written consent form will be signed by each participant before their enrolment and it will be kept in safe custody and personal identification will be kept confidential.
- The project personnel will be trained about the study protocol and data collection before the initiation of the trial.

#### Confidentiality

The subjects will be informed by the investigator that all trial results recorded will be treated in strict confidence. During documentation and analysis of the trial, the individual subject will only be identified by their subject number, whereas the name of the subject and any personal data will come under data protection regulations.

#### Source documents

Source data is all information, original records of clinical findings, observations, or other activities in a clinical trial necessary for the reconstruction and evaluation of the trial. Source data are contained in source documents, i.e., hospital records, clinical and office charts, laboratory notes, memoranda, subjects’ diaries or evaluation check lists, pharmacy dispensing records, recorded data from automated instruments, copies or transcriptions certified after verification as being accurate and complete, subject files and records kept at the pharmacy, at the laboratories and at medico-technical departments involved in the clinical trial.

All information regarding clinical trial will be properly documented, carefully handled, and meticulously stored in order to ensure its accurate interpretation and verification.

#### Case report forms(CRF)

The study case report form is the primary data collection instrument for the study. All the data requested on the CRF will be recorded. All missing data will be explained. If a space on the CRF is left blank because the procedure was not done or the question was not asked, will write “N/D”. If the item is not applicable to the individual case, will write “N/A”. All entries will be printed legibly in black ink. If any entry error has been made, to correct such an error, will draw a single straight line through the incorrect entry and enter the correct data above it. All such changes will be initiated and dated. *Will not erase or white out errors.* For clarification of illegible or uncertain entries, will print the clarification above the item, then initial and date it.

#### Recordkeeping

The original records of subjects will be maintained at the trial site.

#### Study management at site

Trial site will be responsible for setting up an information system to keep track of all subjects screened, enrolled and will maintain a filing system to keep all study records, study protocol, related documentation, and medicine distribution records. The investigator will be responsible for the completeness and accuracy of the study materials.

#### Handling missing data

1. Strategies for handling missing data
  1. Data collection: to minimize missing data, we will implement thorough data collection procedures and follow up regularly with participants. Data checks and reminders will be employed to reduce missing data incidents.
  2. Imputation methods:
    ▪ Multiple imputation: missing data will be addressed using multiple imputation techniques. This involves generating several imputed datasets to account for missing values, analyzing each dataset separately, and then combining the results to reflect the uncertainty due to missing data. This method assumes data are missing at random (MAR).
    ▪ Last observation carried forward (LOCF): for missing data due to non-response at specific time points, LOCF may be used as a supplementary approach, assuming the last observed value is valid in the absence of further data.
  3. Model-based approaches:
    ▪ Mixed-effects models: mixed-effects models will be used to handle missing data by accounting for both fixed and random effects, providing robust estimates despite incomplete data.
    ▪ Maximum likelihood estimation (MLE): MLE will be employed to estimate model parameters, incorporating missing data directly into the estimation process under the assumption of data being missing at random.
  4. Sensitivity analysis: sensitivity analyses will evaluate the impact of different missing data handling methods on study results, assessing the robustness of findings under various assumptions about the missing data.
2. Analysis of non-adherence to intervention
  a Analysis of participants who do not adhere:
    1. Intention-to-treat analysis: participants will be analyzed according to their randomization assignment regardless of adherence. This approach maintains the benefit of randomization and provides an unbiased estimate of treatment effects.
    2. Per-protocol analysis: a per-protocol analysis will be performed for participants who adhere to the intervention as prescribed, assessing the effectiveness under optimal conditions.
    3. Compliance analysis:
      ▪ Adherence rates: descriptive statistics will be used to calculate and characterize adherence rates.
      ▪ Effectiveness among adherers: subgroup analyses will evaluate the effectiveness of the intervention among participants who follow the prescribed treatment.
    4. Instrumental variable analysis: if applicable, instrumental variable analysis may be used to address non-adherence issues by using variables correlated with adherence but not directly with the outcome.
3. Imputation clarification
  a Imputation procedures:
    1. Multiple imputation approach:
      ▪ Creating multiple imputed datasets: several datasets with imputed values will be created based on observed data and assumptions about missing data mechanisms.
      ▪ Analysis of each dataset: each imputed dataset will be analyzed separately using the chosen statistical models.
      ▪ Combining results: results from each imputed dataset will be combined using Rubin’s rules, accounting for within-imputation and between-imputation variability.
    2. Assumptions and validation: the assumptions behind the imputation methods will be validated, and sensitivity analyses will test the robustness of results under different imputation scenarios.
    3. Reporting: the imputation methods and rationale will be clearly reported, including the extent of missing data and the impact of imputation on the findings.

#### Non-enrollment of high-risk patients

Reasons for the non-enrollment of high-risk patients will be documented.

#### To address missing data from enrolled participants

Missing data will be managed using statistical techniques and enhanced data collection methods.

#### Loss of follow-up

Reasons for loss of follow-up will be recorded during the trial to assess impact.

#### Timelines with deliverables

Total study period: 36 months

Trial preparation: 01–03 months (drug supply, IEC approval, staff recruitment, etc.)

Recruitment: 03–33 months (treatment period: 21–25 weeks)

Statistical analysis and preparation of report: 03 months

Outcome analysis will be done after decoding the allocation sequence.

### Statistical analysis

Data will be coded and recorded in MS Excel spreadsheet program. SPSS v23 (IBM Corp.) will be used for data analysis. Descriptive statistics will be expressed in the form of mean/standard deviation and median/IQR for continuous variables, and frequency and percentage for categorical variable. Data will be presented in a graphical form wherever appropriate for data visualization using histograms/box-and-whisker plots/column chart for continuous data and bar chart/pie chart for categorical data. Group comparison for continuously distributed data will be done using independent sample “*t*” test when comparing two groups, and one-way ANOVA when comparing more than two groups. Post hoc pairwise analysis will be performed using Tukey’s HSD test in the case of one-way ANOVA to control for alpha inflation. If data will be found to be non-normally distributed, appropriate non-parametric tests in the form of Wilcoxon test/Kruskal–Wallis test will be used for these comparisons. Chi-squared test will be performed for group comparisons for categorical data. In case the expected frequency in the contingency tables will be found to be < 5 for > 25% of the cells, Fisher’s exact test will be applied instead. Linear correlation between two continuous variables will be explored using Pearson’s correlation (if the data will be normally distributed) and Spearman’s correlation (for non-normally distributed data) and *p* < 0.05 will be considered as statistical significant.

### Ethical issues

**Fig. 2:**
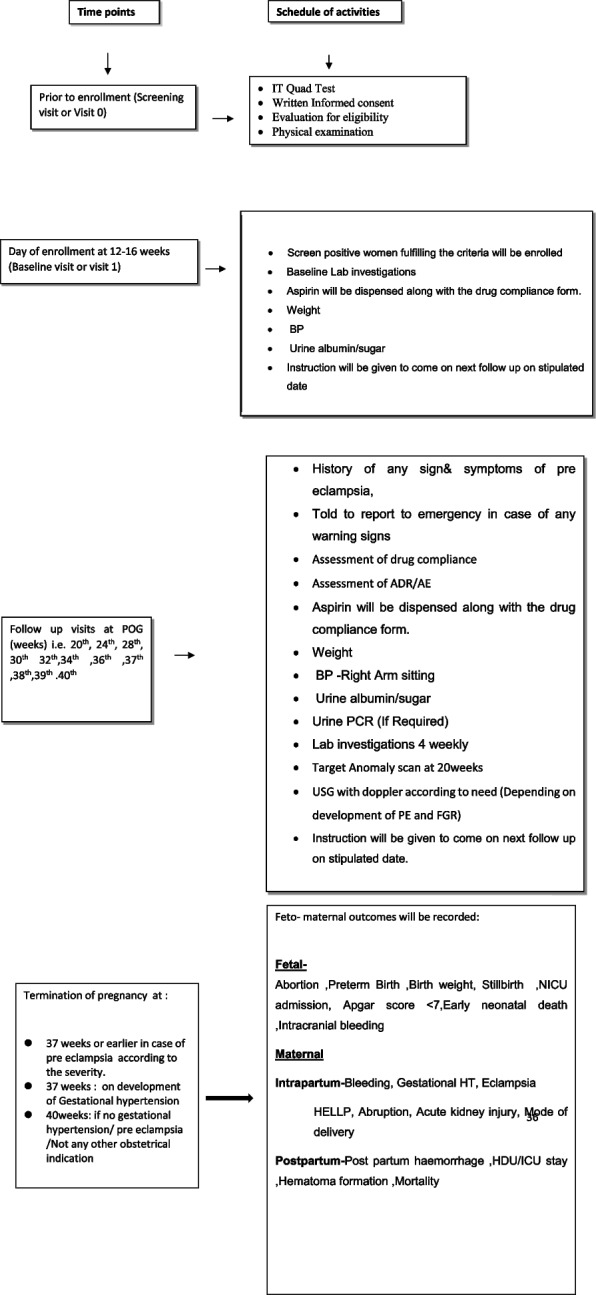
Study schedule

The trial will be registered in CTRI and ethical clearance will be obtained from the Institutional Ethical Committee (IEC) of VMMC & Safdarjung Hospital, New Delhi, before the start of the trial Fig. 2.

## Discussion

In conducting this study, several practical and operational considerations merit discussion. Firstly, ensuring adequate recruitment of high-risk women meeting the inclusion criteria is crucial for achieving the desired sample size and statistical power. Recruitment efforts may need to be intensified, and multiple recruitment sites may be necessary to enhance participant enrollment.

Secondly, maintaining the double-blind nature of the trial presents operational challenges. Strict adherence to blinding protocols, including proper labeling and randomization procedures, is essential to minimize bias in treatment assignment and outcome assessment. Additionally, efforts should be made to ensure that participants, researchers, and clinicians remain blinded throughout the study duration.

Thirdly, monitoring and managing potential adverse events related to aspirin administration are essential aspects of participant safety. Vigilant monitoring for adverse reactions, particularly bleeding complications, is necessary to promptly identify and manage any adverse events that may arise during the study period.

Furthermore, maintaining participant adherence to the assigned aspirin regimen poses practical challenges. Adequate counseling and education regarding the importance of medication adherence may be necessary to promote participant compliance with the prescribed treatment regimen.

Lastly, data collection and management procedures must be meticulously planned and implemented to ensure the accuracy and integrity of study findings. Standardized data collection tools and procedures should be utilized, and robust data management systems should be in place to facilitate efficient data capture, storage, and analysis.

Addressing these practical and operational considerations will be essential for the successful implementation and completion of the study, ultimately ensuring the validity and reliability of the study findings.

## Trial status

The protocol version number for this study is IECVMMC/SJH/Project/2023–12/410, and it was dated 28 February 2023. Screening recruitment for the trial began on 1 May 2024, and it is projected to conclude on 03 March 2026. The trial is registered with the number CTRI/2023/12/060983 registered on 29 December 2023 and has an acknowledgement number of REF/2023/12/076358 dated 29 December 2023.

Total study period: 36 months

Trial preparation: 01–03 months (drug supply, IEC approval, staff recruitment, etc.)

Recruitment: 03–33 months (treatment period: 21–25 weeks)

Statistical analysis and preparation of report: 03 months

Outcome analysis will be done after decoding the allocation sequence.

## Supplementary Information

Additional file 1: Participant Information Sheet(PIS)-English.

Additional file 2: Participant Informed Consent Form.

Additional file 3: Patient’s Proforma.

Additional file 4.

Additional file 5.

## Data Availability

The datasets analyzed during the current study and the statistical code are available from the PI/corresponding author on request. Access to data: access to the final trial dataset is typically granted to the principal investigator. Provisions for ancillary and post-trial care and compensation for harm resulting from trial participation are outlined in the trial protocol. Participants are provided with health insurance.

## Abbreviations

ANC: Antenatal Care
GI: Gastrointestinal
HDU: High dependency unit
HELLP: Hemolysis, elevated liver enzymes, low platelet count
HDP: Hypertensive disorders of pregnancy
HT: Hypertension
ICU: Intensive care unit
NICU: Neonatal intensive care unit
NSAIDs: Nonsteroidal anti-inflammatory drugs
PE: Pre-eclampsia
POG: Period of gestation
SAE: Serious adverse event

## References

[1] Van Doorn R, Mukhtarova N, Flyke IP, Lasarev M, Kim K, Hennekens CH, et al. Dose of aspirin to prevent preterm preeclampsia in women with moderate or high-risk factors: a systematic review and meta-analysis. PLoS ONE. 2021;16(3):e0247782. 10.1371/journal.pone.0247782.PMID:33690642;PMCID:PMC7943022. 33690642 10.1371/journal.pone.0247782 PMC7943022

[2] Sisti G, Fochesato C, Elkafrawi D, Marcus B, Schiattarella A. Is blood pressure 120–139/80-89 mmHg before 20 weeks a risk factor for hypertensive disorders of pregnancy? A meta-analysis. Eur J Obstet Gynecol Reprod Biol. 2023;284:66–75. 10.1016/j.ejogrb.2023.03.011. Epub 2023 Mar 15 PMID: 36934679. 36934679 10.1016/j.ejogrb.2023.03.011

[3] Dhinwa M, Gawande K, Jha N, Anjali M, Bhadoria AS, Sinha S. Prevalence of hypertensive disorders of pregnancy in India: a systematic review and meta-analysis. Journal of Medical Evidence. 2021;2(2):105. 10.4103/JME.JME_168_20.

[4] Magee LA, Sharma S, Nathan HL, Adetoro OO, Bellad MB, Goudar S, et al; CLIP Study Group. The incidence of pregnancy hypertension in India, Pakistan, Mozambique, and Nigeria: a prospective population-level analysis. PLoS Med. 2019 Apr 12;16(4):e1002783. 10.1371/journal.pmed.1002783. PMID: 30978179; PMCID: PMC6461222. 10.1371/journal.pmed.1002783 PMC6461222 30978179

[5] Dandona L, Dandona R, Kumar RK, Kumar GA, Kumar R, Kumar A. Nations within a nation: variations in epidemiological transition across the states of India, 1990–2016 in the Global Burden of Disease Study. Lancet. 2017;390(10111):2437–60. 29150201 10.1016/S0140-6736(17)32804-0 PMC5720596

[6] Nobis PN, Hajong A. Eclampsia in India through the decades. J Obstet Gynaecol India. 2016 Oct;66(Suppl 1):172–6. 10.1007/s13224-015-0807-5. Epub 2016 Jan 8. PMID: 27651598; PMCID: PMC5016424. 10.1007/s13224-015-0807-5 PMC5016424 27651598

[7] Welters SM, de Boer M, Teunissen PW, Hermes W, Ravelli ACJ, Mol BW, et al. Cardiovascular mortality in women in their forties after hypertensive disorders of pregnancy in the Netherlands: a national cohort study. Lancet Healthy Longev. 2023;4(1):e34–42. 10.1016/S2666-7568(22)00292-6. PMID: 36610446. 36610446 10.1016/S2666-7568(22)00292-6

[8] Wu P, Haththotuwa R, Kwok CS, Babu A, Kotronias RA, Rushton C, et al. Preeclampsia and future cardiovascular health: a systematic review and meta-analysis. Circ Cardiovasc Qual Outcomes. 2017;10(2):e003497. 10.1161/CIRCOUTCOMES.116.003497. Epub 2017 Feb 22. PMID: 28228456. 10.1161/CIRCOUTCOMES.116.003497 28228456

[9] Rana S, Lemoine E, Granger JP, Karumanchi SA. Preeclampsia: pathophysiology, challenges, and perspectives. Circ Res. 2019;124(7):1094–112. 10.1161/CIRCRESAHA.118.313276.Erratum.In:CircRes.2020Jan3;126(1):e8. PMID: 30920918. 30920918 10.1161/CIRCRESAHA.118.313276

[10] Brown CE, Casey H, Dominiczak AF, Kerr S, Campbell A, Delles C. Impact of preeclampsia on cardiovascular events: an analysis of the Generation Scotland: Scottish family health study. J Hum Hypertens. 2023;37(8):735–741. 10.1038/s41371-023-00812-2. Epub 2023 Mar 27. PMID: 36973315; PMCID: PMC10403345. 10.1038/s41371-023-00812-2 PMC10403345 36973315

[11] Rolnik DL, Wright D, Poon LC, O’Gorman N, Syngelaki A, de Paco MC, et al. Aspirin versus placebo in pregnancies at high risk for preterm preeclampsia. N Engl J Med. 2017;377(7):613–22. 28657417 10.1056/NEJMoa1704559

[12] Stevens W, Shih T, Incerti D, Ton TG, Lee HC, Peneva D, et al. Short-term costs of preeclampsia to the United States health care system. Am J Obstet Gynecol. 2017;217(3):237–48. 28708975 10.1016/j.ajog.2017.04.032

[13] ACOG Committee Opinion No. 743: Low-dose aspirin use during pregnancy. Obstet Gynecol. 2018;132(1):e44–52. 10.1097/AOG.0000000000002708. PMID: 29939940. 29939940 10.1097/AOG.0000000000002708

[14] Davidson KW, Barry MJ, Mangione CM, Cabana M, Caughey AB, Davis EM, et al. Aspirin use to prevent preeclampsia and related morbidity and mortality: US Preventive Services Task Force recommendation statement. JAMA. 2021;326(12):1186–91. 34581729 10.1001/jama.2021.14781

[15] Hastie R, Tong S, Wikström AK, Sandström A, Hesselman S, Bergman L. Aspirin use during pregnancy and the risk of bleeding complications: a Swedish population-based cohort study. Am J Obstet Gynecol. 2021;224(1):95.e1-95.e12. 10.1016/j.ajog.2020.07.023. Epub 2020 Jul 17 PMID: 32687818. 32687818 10.1016/j.ajog.2020.07.023

[16] Rolnik DL, Nicolaides KH, Poon LC. Prevention of preeclampsia with aspirin. Am J Obstet Gynecol. 2022;226(2S):S1108–19. 10.1016/j.ajog.2020.08.045. Epub 2020 Aug 21 PMID: 32835720. 32835720 10.1016/j.ajog.2020.08.045

[17] Kumar N, Das V, Agarwal A, Pandey A, Agrawal S, Singh A. Pilot interventional study comparing fetomaternal outcomes of 150 mg versus 75 mg aspirin starting between 11 and 14 weeks of pregnancy in patients with high risk of preeclampsia: a randomized control trial. J Obstet Gynaecol India. 2020;70(1):23–29. 10.1007/s13224-019-01277-5. Epub 2019 Sep 20. PMID: 32030002; PMCID: PMC6982625. 10.1007/s13224-019-01277-5 PMC6982625 32030002

[18] Poon LC, Wright D, Rolnik DL, Syngelaki A, Delgado JL, Tsokaki T, et al. Aspirin for evidence-based preeclampsia prevention trial: effect of aspirin in prevention of preterm preeclampsia in subgroups of women according to their characteristics and medical and obstetrical history. Am J Obstet Gynecol. 2017;217(5):585.e1–585.e5. 10.1016/j.ajog.2017.07.038. Epub 2017 Aug 4. Erratum in: Am J Obstet Gynecol. 2018 Feb 1;: PMID: 28784417. 10.1016/j.ajog.2017.07.038 28784417

[19] Chen J, Huai J, Lin L, Li B, Zhu Y, Yang H. Low-dose aspirin in the prevention of pre-eclampsia in China: postpartum hemorrhage in subgroups of women according to their characteristics and potential bleeding risk. Chin Med J (Engl). 2023;136(5):550–5. 10.1097/CM9.0000000000002545.PMID:36914957;PMCID:PMC10106256. 36914957 10.1097/CM9.0000000000002545 PMC10106256

[20] Henderson JT, Vesco KK, Senger CA, Thomas RG, Redmond N. Aspirin use to prevent preeclampsia and related morbidity and mortality: updated evidence report and systematic review for the US Preventive Services Task Force. JAMA. 2021;326(12):1192–206. 10.1001/jama.2021.8551. PMID: 34581730.34581730 10.1001/jama.2021.8551

[21] Xiao Y, Ling Q, Yao M, Gu Y, Lan Y, Liu S, et al. Aspirin 75 mg to prevent preeclampsia in high-risk pregnancies: a retrospective real-world study in China. Eur J Med Res. 2023;28(1):56. 10.1186/s40001-023-01024-7.PMID:36732824;PMCID:PMC9893656. 36732824 10.1186/s40001-023-01024-7 PMC9893656

[22] Wright D, Rolnik DL, Syngelaki A, de Paco MC, Machuca M, de Alvarado M, et al. Aspirin for evidence-based preeclampsia prevention trial: effect of aspirin on length of stay in the neonatal intensive care unit. Am J Obstet Gynecol. 2018;218(6):612.e1-612.e6. 10.1016/j.ajog.2018.02.014. Epub 2018 Mar 2 PMID: 29505771. 29505771 10.1016/j.ajog.2018.02.014

[23] Rolnik DL, Wright D, Poon LCY, Syngelaki A, O'Gorman N, de Paco Matallana C, et al. ASPRE trial: performance of screening for preterm pre-eclampsia. Ultrasound Obstet Gynecol. 2017;50(4):492–495. 10.1002/uog.18816. Epub 2017 Aug 24. Erratum in: Ultrasound Obstet Gynecol. 2017 Dec;50(6):807. PMID: 28741785. 10.1002/uog.18816 28741785

