# Supplementary material for: Comparison of 75 mg versus 150 mg aspirin for the prevention of preterm preeclampsia in high-risk women at a tertiary level hospital: study protocol for a randomized double-blind clinical trial
Source: Trials. 2024 Oct 15;25:679. doi: 10.1186/s13063-024-08520-z (PMC11476763; doi:10.1186/s13063-024-08520-z)
Supplement: Supplementary file 1 — Additional file 1: Participant Information Sheet(PIS)-English. [file 13063_2024_8520_MOESM1_ESM.docx]

**Appendix I: Participant Information Sheet(PIS)-English**

You are participating into a study on: “Comparison of 75 mg versus 150 mg Aspirin for prevention of preterm preeclampsia in high risk women at Tertiary level hospital-A Randomized double blind clinical trial.” You will be screened for preterm PE using first trimester Quadruple testing. Your blood sample (3ml) will be taken for first trimester quadruple marker test between 11-13 ^+ 6^ weeks. If the risk for preterm PE is 1:100 or greater, it will be labeled as Screen positive or high risk and you will be started on aspirin and will be followed till delivery to look for outcome.

The data generated from the study will help to determine the optimal dose of Aspirin ( 75mg versus 150 mg) necessary for development of pre-eclampsia in high risk women. Low dose aspirin has a good maternal and fetal safety profile with few risks of intrapartum and postpartum hemorrhage^.^

In this study, you/your patient will not be subjected to any additional risk/research related injury. During the study you will be asked some question related to the study. No additional cost will be incurred to you/your patient as a part of research.

Participation in this study in voluntary. You/your patient will have the right to withdraw any time from the study without any penalty or loss of benefits otherwise entitled to. All patient records and information will be kept confidential.

In case you have any questions related to the study, you can contact:

Dr. Upma Saxena: 9971460540

(Signature/ Left thumb impression)

Participant Name: --------------

Date:

Place:

**Participant Information Sheet (PIS)-Hindi**

**परिशिष्ट 1 : प्रतिभागी सूचना पत्रक**

आप " **तृतीयक स्तर के अस्पताल में गंभीर जोखिम वाली महिलाओं के समय से पहले होने वाले बच्चों में प्रीक्लेम्पसिया की रोकथाम के लिए 75 मिलीग्राम बनाम 150 मिलीग्राम एस्पिरिन दवा की तुलना – एक यादृच्छिक दोहरा विचारहीन नैदानिक परीक्षण** " नामक अध्ययन में भाग ले रहे हैं। पहले तिमाही क्वाड्रपल परीक्षण का उपयोग करते हुए आपका प्रीटर्म पीई (प्री-एक्लेम्पसिया) की जांच की जाएगी। 11-13 + 6 सप्ताह के बीच पहली तिमाही के क्वाड्रपल मार्कर परीक्षण के लिए आपके रक्त का नमूना (3 मि.ली.) लिया जाएगा। यदि प्रीटर्म पीई का जोखिम 1:100 या इससे अधिक है, तो इसे स्क्रीन पॉजिटिव या उच्च जोखिम के रूप में चिन्हीत किया जाएगा और आपको एस्पिरिन दवा देना शुरू कर दिया जाएगा और परिणाम देखने के लिए डिलीवरी तक निगरानी की जाएगी।

अध्ययन से उत्पन्न डेटा उच्च जोखिम वाली महिलाओं में प्री-एक्लम्पसिया के विकास के लिए आवश्यक एस्पिरिन (75 मि.ग्रा. बनाम 150 मि.ग्रा.) की सही समझी जाने वाली खुराक निर्धारित करने में मदद करेगा। प्रसव होने के समय और प्रसव हो जाने के बाद रक्तस्राव के कुछ जोखिम के साथ एस्पिरिन की कम खुराक मातृ एवं भ्रूण सुरक्षा के प्रोफ़ाइल के लिए अच्छी होती हैं।

इस अध्ययन में, आपको/आपके मरीज को किसी भी अतिरिक्त जोखिम/अनुसंधान संबंधी चोट का सामना नहीं करना पड़ेगा। अध्ययन के दौरान आपसे अध्ययन से संबंधित कुछ प्रश्न पूछे जायेंगे। शोध के भाग के रूप में आपको/आपके मरीज़ को कोई अतिरिक्त लागत का भुगतान नहीं किया जाएगा।

इस अध्ययन में भागीदारी स्वैच्छिक है। आपको/आपके मरीज को किसी भी समय बिना किसी जुर्माने या लाभों की हानि हुए बगैर अध्ययन से हटने का अधिकार होगा, जिसका रोगी अन्यथा हकदार है। मरीज के सभी रिकॉर्ड और जानकारी गोपनीय रखी जाएगी।

यदि आपके पास अध्ययन से संबंधित कोई प्रश्न है, तो आप संपर्क कर सकते हैं:

**डॉ. उपमा सक्सैना : 9971460540**

..........................................................

(हस्ताक्षर / बाएं अंगूठे का निशान)

प्रतिभागी का नाम : ----------------

तारीख : ----------------

जगह : ---------------
