# Supplementary material for: Comparison of 75 mg versus 150 mg aspirin for the prevention of preterm preeclampsia in high-risk women at a tertiary level hospital: study protocol for a randomized double-blind clinical trial
Source: Trials. 2024 Oct 15;25:679. doi: 10.1186/s13063-024-08520-z (PMC11476763; doi:10.1186/s13063-024-08520-z)
Supplement: Supplementary file 2 — Additional file 2: Participant Informed Consent Form. [file 13063_2024_8520_MOESM2_ESM.docx]

**Appendix II: Participant Informed Consent Form**

Protocol/ Study number:

Participant identification number for this trial:

Title of project: Comparison of 75 mg versus 150 mg Aspirin for prevention of preterm pre-eclampsia in high risk women at Tertiary level hospital-A Randomized double blind clinical trial

Name of Principal Investigator: Dr Upma Saxena Tel No.: 9971460540

The contents of the participant information sheet dated…..that was provided have been read carefully by me/ explained in detail to me, in a language that I comprehend, and I have fully understood the contents. I confirm that I have had the opportunity to ask questions and all my questions have been answered to my full satisfaction.

The nature and purpose of the study and its potential risks/ benefits and expected duration of the study, and other relevant details have been explained to me in detail.

I understand that my participation in this study is voluntary and I am free to withdraw my participation at any time, without any medical care or legal rights being affected.

I understand that the ethics committee and the regulatory authorities will not need my permission to look at my health records both in respect of the current study and any further research that may be conducted in relation to it, even if I withdraw from the study. I agree to this access. However, I understand that my identity will not be exposed in any information released to third parties or published.

I, _________ daughter/wife of ________ resident of ________ do hereby give my free and voluntary consent for participation in the study. I also agree to allow use of data arising from this study for scientific purposes without disclosing my identity.

(Signature/thumb impression) Date:

Place:

Name of participant:

Son/Daughter/ Spouse of Participant:

Complete Postal address:

This is to certify that the above consent has been obtained in my presence.

….………………… Date:

Signature of Principal Investigator Place:

1. Witness-1 2. Witness-2

….………………. ………………..

Signature Signature

Name: Name:

Address: Address:

**परिशिष्ट 2**

**प्रतिभागी सूचित सहमति प्रपत्र**

प्रोटोकॉल / अध्ययन संख्या : ..........................

इस परीक्षण के लिए प्रतिभागी पहचान संख्या : .............................

**परियोजना का शीर्षक : तृतीयक स्तर के अस्पताल में गंभीर जोखिम वाली महिलाओं के समय से पहले होने वाले बच्चों में प्रीक्लेम्पसिया की रोकथाम के लिए 75 मिलीग्राम बनाम 150 मिलीग्राम एस्पिरिन दवा की तुलना – एक यादृच्छिक दोहरा विचारहीन नैदानिक परीक्षण।**

प्रधान अन्वेषक का नाम : डॉ. उपमा सक्सेना टेलीफोन नंबर : 9971460540

इस प्रतिभागी सूचना पत्र की जानकारी दिनांक ................................. को प्रदान की गई हैं, जिसे मैंने ध्यानपूर्वक पढ़ा है/मुझे मेरी समझ में आने वाली भाषा में विस्तार से समझाया गया है, और मैंने सामग्री को पूरी तरह से समझ लिया है। मैं पुष्टि करता/ती हूं कि मुझे प्रश्न पूछने का अवसर मिला है और मेरे सभी प्रश्नों का मेरी पूरी संतुष्टि के साथ उत्तर दिया गया है।

अध्ययन की प्रकृति और उद्देश्य और इसके संभावित जोखिम/लाभ और अध्ययन की अपेक्षित अवधि और अन्य प्रासंगिक विवरण मुझे विस्तार से समझाए गए हैं।

मैं समझता/ती हूं कि इस अध्ययन में मेरी भागीदारी स्वैच्छिक है और मैं किसी भी चिकित्सा देखभाल या कानूनी अधिकारों को प्रभावित किए बिना, किसी भी समय अपनी भागीदारी वापस लेने के लिए स्वतंत्र हूं।

मैं समझता/ती हूं कि नैतिक समिति और नियामक अधिकारियों को वर्तमान अध्ययन और इससे संबंधित किए जाने वाले किसी भी आगे के शोध के बारे में मेरे स्वास्थ्य रिकॉर्ड को देखने के लिए मेरी अनुमति की आवश्यकता नहीं होगी, भले ही मैं अध्ययन से हट जाऊं। मैं इस पहुंच से सहमत हूं। हालाँकि, मैं समझता/ती हूँ कि तीसरे/अन्य पक्ष को जारी किये जाने या प्रकाशित किसी भी जानकारी में मेरी पहचान का उजागर नहीं किया जाएगा।

मैं, ________ बेटी / पत्नी ________ की, ________ की निवासी, इसके द्वारा मैं अध्ययन में भाग लेने के लिए अपनी स्वतंत्र रूप से और स्वैच्छिक सहमति देती हूं। मैं अपनी पहचान उजागर किए बिना वैज्ञानिक उद्देश्यों के लिए इस अध्ययन से प्राप्त डेटा के उपयोग की अनुमति देने के लिए भी सहमत हूं।

(हस्ताक्षर/अंगूठे का निशान) दिनांक : ________ जगह : ________

प्रतिभागी का नाम : ________________

प्रतिभागी का बेटा/बेटी/पति/पत्नी : ________________

पूरा डाक पता : ________________

प्रमाणित किया जाता है कि उपरोक्त सहमति मेरी उपस्थिति में प्राप्त की गई है।

………………… तारीख : ________________

प्रधान अन्वेषक के हस्ताक्षर स्थान : ________________

1. गवाह-1 2. गवाह-2

…………………. ………………..

हस्ताक्षर ________ हस्ताक्षर ________

नाम ________ नाम ________

पता ________ पता ________
