# Supplementary material for: Comparison of 75 mg versus 150 mg aspirin for the prevention of preterm preeclampsia in high-risk women at a tertiary level hospital: study protocol for a randomized double-blind clinical trial
Source: Trials. 2024 Oct 15;25:679. doi: 10.1186/s13063-024-08520-z (PMC11476763; doi:10.1186/s13063-024-08520-z)
Supplement: Supplementary file 3 — Additional file 3: Patient’s Proforma. [file 13063_2024_8520_MOESM3_ESM.docx]

**Appendix III: Patient’s Proforma**

**PATIENT PROFORMA SHEET**

S.no : MRD no:

Name: Age: Phone no:

Address: Education:

No of ANC visits:

Height: Weight: BMI:

BP: Pulse

History And Examination

Menstrual History: LMP: EDD:

Cycle: Regular/Irregular

Obstetrics History:

| G | P | L | A |
| --- | --- | --- | --- |
|  |  |  |  |

PAST HISTORY OF PE:

MEDICAL HISTORY:

SURGICAL HISTORY:

ALLERGY HISTORY( e.g. Aspirin):

FAMILY HISTORY:

RACE:

EXAMINATION

General

| Date |  |  |  |  |  |  |  |  |
| --- | --- | --- | --- | --- | --- | --- | --- | --- |
| POG |  |  |  |  |  |  |  |  |
| TEMP  PULSE |  |  |  |  |  |  |  |  |
| BP |  |  |  |  |  |  |  |  |
| PALLOR |  |  |  |  |  |  |  |  |
| ICTERUS |  |  |  |  |  |  |  |  |
| EDEMA |  |  |  |  |  |  |  |  |
| RR |  |  |  |  |  |  |  |  |
| Systemic |  |  |  |  |  |  |  |  |
| CNS |  |  |  |  |  |  |  |  |
| CVS |  |  |  |  |  |  |  |  |
| Respiratory system |  |  |  |  |  |  |  |  |

P/A Examination

| Date |  |  |  |  |  |  |  |  |
| --- | --- | --- | --- | --- | --- | --- | --- | --- |
| POG |  |  |  |  |  |  |  |  |
| Fundal Height |  |  |  |  |  |  |  |  |
| FHS |  |  |  |  |  |  |  |  |
| Presentation |  |  |  |  |  |  |  |  |
| Uterus |  |  |  |  |  |  |  |  |
| PS/PV ( if performed) |  |  |  |  |  |  |  |  |

**INVESTIGATIONS**

First trimester Quadruple Marker test:

Screen positive/ Negative

| Date |  |  |  |  |  |  |  |
| --- | --- | --- | --- | --- | --- | --- | --- |
| POG |  |  |  |  |  |  |  |
| CBC with platelet count |  |  |  |  |  |  |  |
| P/S |  |  |  |  |  |  |  |
| Coagulation Profile |  |  |  |  |  |  |  |
| Urine microscopy |  |  |  |  |  |  |  |
| OGTT |  |  |  |  |  |  |  |
| LFT |  |  |  |  |  |  |  |
| KFT |  |  |  |  |  |  |  |
| Urine protein |  |  |  |  |  |  |  |
| TSH |  |  |  |  |  |  |  |
| Any other ( specify) |  |  |  |  |  |  |  |

USG

| Date |  |  |  |  |  |  |  |
| --- | --- | --- | --- | --- | --- | --- | --- |
| POG |  |  |  |  |  |  |  |
| USG |  |  |  |  |  |  |  |

PRIMARY OUTCOME

Preterm Pre-eclampsia YES/NO

Delivery Details

1. Term/ Preterm/ POG at the time of delivery:
2. Mode of delivery: normal vaginal/ vacuum/ ventouse/ forceps/ LSCS/ Any other
3. Any complications ( specify):
4. Blood Transfusion: yes/ no.

If yes, no. of units (specify)

Baby Details

1. Stillborn/ Live/ neonatal/ NICU admission
2. Weight
3. Sex
4. Time
5. APGAR score

SECONDARY OUTCOMES

Maternal

| Bleeding ( specify site) |  |  |
| --- | --- | --- |
| Gestational HTN |  |  |
| Eclampsia |  |  |
| Abruption |  |  |
| HELLP |  |  |
| Acute Kidney Injury |  |  |
| Mode of delivery |  |  |
| Post partum hemorrhage |  |  |
| HDU/ICU stay |  |  |
| Hematoma formation |  |  |
| Any other ( specify) |  |  |
| Mortality |  |  |

Fetal

| Abortion |  |  |
| --- | --- | --- |
| Preterm Birth |  |  |
| Birth weight |  |  |
| Still Birth |  |  |
| NICU admission |  |  |
| APGAR score |  |  |
| Early neonatal death |  |  |
| Intracranial hemorrhage |  |  |
| Any other ( specify) |  |  |
