# Supplementary material for: Comparison of 75 mg versus 150 mg aspirin for the prevention of preterm preeclampsia in high-risk women at a tertiary level hospital: study protocol for a randomized double-blind clinical trial
Source: Trials. 2024 Oct 15;25:679. doi: 10.1186/s13063-024-08520-z (PMC11476763; doi:10.1186/s13063-024-08520-z)
Supplement: Supplementary file 5 — Additional file 5. [file 13063_2024_8520_MOESM5_ESM.htm]

| US Words[54] | UKise Words[7] | Ukize Words[25] |
| --- | --- | --- |
| randomized[15] | Acknowledgement[1] | randomized[15] |
| Gynecology[8] | i.e.[15] | emphasizing[1] |
| Center[1] | analyses[4] | Acknowledgement[1] |
| fetal[10] | e.g.[1] | i.e.[15] |
| emphasizing[1] | enrolment[8] | individualized[1] |
| practice[2] | physiological[1] | analyses[4] |
| Fetal[4] | acknowledgement[1] | optimize[1] |
| i.e.,[15] |  | e.g.[1] |
| focused[1] |  | Randomization[2] |
| individualized[1] |  | randomization[11] |
| in future[1] |  | enrolment[8] |
| program[3] |  | minimizes[1] |
| hemorrhage[3] |  | specialized[2] |
| among[4] |  | authorized[2] |
| cesarean[1] |  | physiological[1] |
| While[3] |  | personalized[1] |
| while[1] |  | fertilization[1] |
| focusing[1] |  | emphasized[1] |
| optimize[1] |  | minimize[2] |
| e.g.,[1] |  | characterize[1] |
| labeled[2] |  | visualization[1] |
| hemophilia[1] |  | Standardized[1] |
| Fetus[1] |  | utilized[1] |
| Randomization[2] |  | acknowledgement[1] |
| randomization[11] |  | conceptualized[1] |
| Labeling[1] |  |  |
| checks[2] |  |  |
| minimizes[1] |  |  |
| enrollment[11] |  |  |
| specialized[2] |  |  |
| Enrollment[1] |  |  |
| authorized[2] |  |  |
| Counseling[1] |  |  |
| counseled[1] |  |  |
| counseling[3] |  |  |
| check[4] |  |  |
| personalized[1] |  |  |
| analyzed[4] |  |  |
| fertilization[1] |  |  |
| color[1] |  |  |
| emphasized[1] |  |  |
| edema[2] |  |  |
| hemoglobin[1] |  |  |
| labeling[2] |  |  |
| minimize[2] |  |  |
| analyzing[1] |  |  |
| characterize[1] |  |  |
| visualization[1] |  |  |
| Standardized[1] |  |  |
| utilized[1] |  |  |
| conceptualized[1] |  |  |
| neighboring[2] |  |  |
| programs[1] |  |  |
| center[1] |  |  |

| Hyphenated Words | With Hyphen | Without Hyphen | Closed up |
| --- | --- | --- | --- |
| high-risk | 22 | 4 | 0 |
| double-blind | 8 | 1 | 0 |
| Pre-eclampsia | 4 | 0 | 15 |
| long-term | 6 | 0 | 0 |
| pre-eclampsia | 52 | 0 | 15 |
| Screen-positive | 2 | 2 | 0 |
| Feto-maternal | 1 | 0 | 5 |
| evidence-based | 3 | 0 | 0 |
| Registry-India | 1 | 0 | 0 |
| High-risk | 1 | 4 | 0 |
| Evidence-based | 1 | 0 | 0 |
| anti-inflammatory | 1 | 0 | 0 |
| PEPre-eclampsia | 1 | 0 | 0 |
| cross-sectional | 1 | 0 | 0 |
| short-term | 3 | 0 | 0 |
| follow-up | 18 | 2 | 0 |
| end-stage | 1 | 0 | 0 |
| double-edged | 1 | 0 | 0 |
| pre-existing | 1 | 0 | 0 |
| screen-positive | 8 | 2 | 0 |
| First-trimester | 2 | 4 | 0 |
| PAPP-A | 5 | 0 | 0 |
| low-dose | 7 | 0 | 0 |
| cost-effective | 1 | 0 | 0 |
| Low-dose | 4 | 0 | 0 |
| meta-analyses | 1 | 0 | 0 |
| bleeding-related | 1 | 0 | 0 |
| small-for | 1 | 0 | 0 |
| gestational-age | 1 | 5 | 0 |
| placebo-controlled | 1 | 0 | 0 |
| feto-maternal | 1 | 0 | 5 |
| objective-wise | 1 | 0 | 0 |
| sub-headings | 1 | 0 | 0 |
| first-trimester | 2 | 4 | 0 |
| anti-platelet | 1 | 0 | 0 |
| computer-generated | 3 | 0 | 0 |
| two-armed | 1 | 0 | 0 |
| day-to | 3 | 1 | 0 |
| pre-generated | 1 | 0 | 0 |
| non-adherence | 4 | 0 | 0 |
| o-solving | 1 | 0 | 0 |
| crown-rump | 1 | 0 | 0 |
| Afro-Caribbean | 1 | 0 | 0 |
| pregnancy-associated | 1 | 0 | 0 |
| protein-A | 1 | 0 | 0 |
| trial-related | 2 | 0 | 0 |
| pre-pregnancy | 1 | 0 | 0 |
| four-weekly | 2 | 0 | 0 |
| two-weekly | 2 | 0 | 0 |
| VMMC-Safdarjung | 1 | 0 | 0 |
| GMP-certified | 1 | 0 | 0 |
| new-onset | 1 | 1 | 0 |
| New-onset | 2 | 1 | 0 |
| reagent-strip | 1 | 0 | 0 |
| high-level | 2 | 0 | 0 |
| decision-making | 1 | 0 | 0 |
| semi-annually | 1 | 0 | 0 |
| Risk-based | 1 | 0 | 0 |
| quality-assured | 1 | 0 | 0 |
| medico-technical | 1 | 0 | 0 |
| non-response | 1 | 0 | 0 |
| Model-based | 1 | 0 | 0 |
| Mixed-effects | 1 | 0 | 0 |
| mixed-effects | 1 | 0 | 0 |
| Intention-to | 1 | 0 | 0 |
| Per-protocol | 1 | 1 | 0 |
| per-protocol | 1 | 1 | 0 |
| within-imputation | 1 | 0 | 0 |
| between-imputation | 1 | 0 | 0 |
| Non-enrollment | 1 | 0 | 0 |
| non-enrollment | 1 | 0 | 0 |
| box-and | 1 | 0 | 0 |
| one-way | 2 | 0 | 0 |
| non-normally | 2 | 0 | 0 |
| non-parametric | 1 | 0 | 0 |
| Chi-squared | 1 | 0 | 0 |
| tertiary-level | 1 | 1 | 0 |
| multi-disciplinary | 1 | 0 | 0 |
| Co-principal | 1 | 0 | 0 |
| co-PIs | 1 | 0 | 0 |
| co-investigator | 1 | 0 | 0 |
| co-PI | 1 | 0 | 0 |
| Co-PI | 1 | 0 | 0 |
| investigator-initiated | 1 | 0 | 0 |
| E-office | 1 | 0 | 0 |
| post-trial | 1 | 0 | 0 |
